# Genotyping and antimicrobial resistance of *Streptococcus uberis* isolated from bovine clinical mastitis

**DOI:** 10.1371/journal.pone.0223719

**Published:** 2019-10-22

**Authors:** Tiago Tomazi, Gustavo Freu, Bruna Gomes Alves, Antonio Francisco de Souza Filho, Marcos Bryan Heinemann, Marcos Veiga dos Santos

**Affiliations:** 1 Department of Animal Production and Nutrition, Milk Quality Research Laboratory (Qualileite), University of São Paulo, Pirassununga, Brazil; 2 Department of Population Medicine and Diagnostic Sciences, School of Veterinary Medicine, Cornell University, Ithaca, New York, United States of America; 3 Department of Preventive Veterinary Medicine and Animal Health, Laboratory of Bacterial Zoonosis, University of São Paulo, São Paulo, Brazil; Tokat Gaziosmanpasa University, TURKEY

## Abstract

A genotypic characterization of *Streptococcus uberis* isolated from clinical mastitis (CM) in dairy cows, and the association of *Strep*. *uberis* genotypes and antimicrobial susceptibility (AMS) was performed. A total of 89 isolates identified as *Strep*. *uberis* from 86 dairy cows with CM in 17 dairy herds of Southeastern Brazil were genotyped using random amplified polymorphic DNA (RAPD) analysis. After genotyping, two clusters (I and II) were created according to RAPD types. A commercial broth microdilution test was used to determine the susceptibility of *Strep*. *uberis* isolates to 8 antimicrobials (ampicillin, ceftiofur, cephalothin, erythromycin, penicillin, penicillin+novobiocin, pirlimycin and tetracycline). For each antimicrobial, we determined the minimal inhibitory concentrations that inhibit 50% (MIC_50_) and 90% (MIC_90_) of *Strep*. *uberis* strains. Differences in AMS among genotypic clusters were evaluated using mixed regression models. Overall, a great polymorphism (56 RAPD-types) was found among *Strep*. *uberis* isolates, although a higher genetic similarity (based on the PCR bands features) was observed within herds after genotypic clustering. No differences in AMS were observed among clusters. *Strep*. *uberis* isolated from bovine CM were resistant to most antimicrobials, with the exception of cephalothin and penicillin+novobiocin.

## Introduction

*Streptococcus uberis* is one of the main causes of clinical mastitis (CM) worldwide [1]. This pathogen is considered an important barrier to control of mastitis in dairy cattle because its epidemiology is not completely understood [2]. While the environment seems to be the main reservoir of *Strep*. *uberis*, further molecular studies provided evidence that contagious transmission also occurs [3–5].

Mastitis prevention programs have focused on reducing the rate of new intramammary infections (IMI; [1]). Management practices such as adequate milking procedures and maintenance of cow hygiene were associated with a reduction of CM [6, 7]. However, despite prevention efforts, CM cannot be eradicated and most affected cows in Brazilian dairy herds are treated with antimicrobials regardless to the isolation and identification of the mastitis-causing pathogen [8]. Antimicrobial treatment was reported in 97% of 5,457 quarter-cases of clinical mastitis occurred in 20 dairy herds. In addition, combination therapy (i.e., association of intramammary and systematically administered antimicrobials) was reported for 64.4% of treatments at the cow-level [8]. Treatment of CM along with blanket dry cow therapy were described as the major causes for antimicrobial consumption in dairy herds [9,10]. The overuse of antimicrobials in livestock may be a contributing factor to the increase of resistance of bacteria to antimicrobials used in human and veterinary medicine [11,12]. In Brazil, antimicrobials used in veterinary medicine are still legally purchased without prescription. This factor could facilitate the indiscriminate use of antibiotics in dairy herds and potentially increase antimicrobial resistance of mastitis causing pathogens.

Genotyping of *Strep*. *uberis* was reported in several countries [4, 13, 14]. However, despite the importance of *Strep*. *uberis* as cause of CM in Brazil [8], no recent investigation has genotyped isolates recovered from CM in this country. The evaluation of diversity among *Strep*. *uberis* isolates could provide information on the potential transmission behavior, and perhaps, on the association between genotypes and the susceptibility profile.

Compared to traditional techniques for microbiological identification (i.e., morphological and biochemical tests), molecular methods have greater discriminatory power and reproducibility. For this reason, genotyping assays have contributed with the knowledge on mastitis epidemiology, including those caused by *Streptococcus* spp. [15–18]. Several DNA fingerprinting methods have been described for genotyping *Strep*. *uberis* isolated from bovine mastitis, which includes restriction fragment length polymorphism (RFLP; [19]), multilocus sequence typing (MLST; [20]) and pulsed-field gel electrophoresis (PFGE; [21]). Among the available molecular typing methods, random amplified polymorphic DNA (RAPD) has been used effectively for molecular characterization of several bacterial species, including *Strep*. *uberis* [2, 22]. RAPD is an easy to perform and inexpensive strain typing method with sufficient discriminatory power for the evaluation of genotypic diversity of *Strep*. *uberis* causing mastitis [2]. However, the main limitation of RAPD is the poor reproducibility of fingerprints, as minimal changes in the PCR conditions can lead to differences in the results.

Antimicrobial resistance has been described for *Strep*. *uberis* identified in CM cases in other countries [23, 24]. However, considering that antimicrobial resistance can vary between regions and even within the same region, it is important to constantly monitor the susceptibility of microorganisms to antimicrobials used for treatment of mastitis in different areas [25]. No study conducted in Brazil has genotyped isolates of *Strep*. *uberis* recovered from CM and determined the antimicrobial susceptibility (AMS) profile at the genetic level. Therefore, the objectives of this study were to: (1) characterize the genotypic diversity of *Strep*. *uberis* isolated from cases of CM in dairy cows and, (2) determine the association between *Strep*. *uberis* genotypes and AMS.

## Material and methods

### Ethics statement

The study was approved by the Ethics Committee on Animal Use of the School of Veterinary Medicine and Animal Science of University of São Paulo (registration code: CEUA 2994060214). All experimental procedures and the care of cows were in strict accordance with the rules issued by the Brazilian National Council for Control of Animal Experimentation (CONCEA; Law 11.794 of October 8, 2008, Decree 6899 of July 15, 2009).

### Isolates of *Streptococcus uberis*

Isolates were selected from a collection of bacteria identified during a prospective epidemiological survey evaluating the etiology and risk factors associated with CM in 20 dairy herds of Southeastern Brazil [26]. The overall mean of daily milk production per cow among herds was 22.7 kg (SD = 5.7). Holstein was the predominant breed among herds (n = 15), although four herds raised Gyr or Gyr × Holstein crossbreds (also called Girolando), and one herd raised only Jersey cattle. Five herds housed their lactating cows in compost bedded pack barns (CBPB), five in free stalls and ten in paddocks. The CBPB is a housing system characterized by an open resting area (free of stalls or partitions) and bedded with organic materials (e.g., sawdust), which must be mechanically stirred on a regular basis [27]. The paddock housing system was defined as an open area surrounded by fences or rails with or without pasture for grazing. Of herds housing cows in free stalls, three (A, D and N) had facilities with deep sand beds, and two had rubber cow mattresses covered with a layer of organic material (i.e.; coffee barks in herd M, and wood shavings in herd K). All herds milked their cows in parallel or herringbone milking parlors, except for herd K that had a rotary milking system.

During the aforementioned study [26], milk samples were collected from quarters with abnormal milk, accompanied or not by other clinical symptoms by trained farm personnel using the following procedures: (1) affected teat were dipped in a pre-milking disinfectant solution; (2) after 30 seconds, the teat was wiped with a disposable paper towel; (3) the first 3–4 milk streams were discarded and the teat end was scrubbed using a gauze soaked in alcohol 70%; (4) milk samples were collected into a sterile 15-mL tube. Samples were stored in the farm (approximately -20°C) and transported to the laboratory in isothermal boxes with ice during visits performed by researchers every 14–30 days. Severity of CM was recorded as mild (alteration of milk appearance), moderate (alteration of milk appearance associated with inflammatory symptoms in the mammary gland), and severe (presence of systemic symptoms) [28].

A total of 256 *Strep*. *uberis* were identified in 4,212 quarter milk samples collected from 5,957 CM cases occurred in 2,637 cows (Table 1). Of cows identified with CM, 48.6% had more than one case of the disease [26]. Microbiological identification was based on the National Mastitis Council guidelines [29]. *Strep*. *uberis* were identified as catalase-negative Gram-positive cocci, with positive or negative Christie-Atkins-Munch-Peterson (CAMP) reaction, positive reaction for esculin activity, and negative reaction in the bile-esculin agar test. Of isolates identified as *Strep*. *uberis*, 193 (75.4%) were cryopreserved (-80°C) in sterile tubes containing brain heart infusion broth (BBL-Becton Dickinson and Co., Le Point de Claix, France) supplemented with 10% glycerin until further analysis.

**Table 1:** Descriptive data at the herd level, absolute frequency of CM (overall and caused by *Streptococcus uberis*), and number of strains selected for genotyping and antimicrobial susceptibility testing.

Herd	Housing^1^	Herd size^2^	Study period	CM^3^	*Number of Strep*. *uberis isolates*			
					Total^4^	Cryop.^5^	Conf.^6^	Select.^7^
A	FS	1470 (52)	Jul/14 –Jul/15	379	50	40	9	9
B	CBPB	184 (25)	Apr/14 –Apr/15	178	9	5	2	2
C	PD	68 (5)	May/14 –Apr/15	36	0	0	0	0
D	FS	165 (11)	Apr/14 –Apr/15	192	20	17	15	13
E	PD	371 (24)	Apr/14 –Apr/15	370	31	28	20	15
F	PD	253 (13)	Mar/14 –Apr/15	212	15	12	9	9
G	PD	77 (15)	Apr/14 –Nov/14	40	1	1	0	0
H	PD	71 (9)	Feb/15 –Jan/16	90	8	6	5	5
I	CBPB	167 (11)	May/14 –Apr/15	356	22	15	5	5
J	CBPB	120 (10)	Apr/14 –Apr/15	69	3	3	2	2
K	FS	313 (7)	Mar/14 –Apr/15	299	6	5	1	1
L	PD	194 (7)	May/14 –Apr/15	280	38	29	21	16
M	FS	586 (17)	Dec/14 –Dec/15	1208	33	12	3	3
N	FS	55 (7)	Apr/14 –Jun/15	76	4	4	1	1
O	PD	46 (3)	Apr/14 –Mar/15	44	4	4	3	3
P	PD	36 (1)	Apr/14 –Apr/15	68	4	4	2	2
Q	CBPB	75 (12)	May/14 –Apr/15	212	4	4	1	1
R	PD	22 (3)	Apr/14 –Mar/15	13	1	1	1	1
S	PD	55 (7)	Jun/14 –May/15	50	2	2	0	0
T	CBPB	46 (4)	Apr/14 –Apr/15	41	1	1	1	1
Overall		219 (318)	Mar/14 –Jan/16	4,212	256	193	101	89

^1^Housing system of herds from which the *Strep*. *uberis* isolates were selected. FS (Free stall); CBPB (Compost Bedded Pack Barn, housing system composed by a resting area bedded with organic materials, which must be mechanically stirred on a regular basis); PD (Paddocks, housing system defined as an open area surrounded by fences or rails with or without pasture for grazing.

^2^Herd size—Average number of lactating cows (standard deviation in parenthesis) per herd during the study period.

^3^Absolute frequency (n) of clinical mastitis cases identified during the study period regardless of causing-pathogen.

^4^Number of clinical mastitis cases with identification of *Streptococcus uberis* (*SU*) in the bacteriological culture.

^5^Number of isolates cryopreserved during the study period.

^6^Number of isolates that were re-cultivated and confirmed as *Strep*. *uberis* using MALDI-TOF MS.

^7^Number of isolates selected for strain typing and AMS testing.

For the present study, all cryopreserved bacteria were thawed and recultured on trypticase soy agar (BBL-Becton Dickinson and Co., Le Point de Claix, France) supplemented with 5% of bovine blood. Matrix-assisted laser desorption ionization–time of flight mass spectrometry (MALDI-TOF MS) was used to confirm the isolates identification at the species level as previously described [30]. Only isolates identified as *Strep*. *uberis* and with MALDI scores >2.0 were eligible for selection. Therefore, 92 of 193 cryopreserved bacteria were not eligible to be selected in this study because of the following reasons: 45 (23.3%) were identified as other streptococci or *Streptococcus*-like bacteria, 27 (14.0%) were contaminated samples (i.e., presence of colonies with different phenotypic features), 15 (7.8%) had no growth during re-cultivation, and 5 (2.6%) were not identified (i.e., no peaks found) using MALDI-TOF MS. Thereby, only 17 of 20 enrolled herds had isolates submitted for strain typing and antimicrobial susceptibility. One herd had no isolation of *Strep*. *uberis* (herd C), while isolates from the other two herds (G and S) were not confirmed as *Strep*. *uberis* using MALDI-TOF MS (Table 1).

A total of 89 strains were selected because we aimed to evaluate all of them in the same batch to avoid potential variations in the molecular results associated with the RAPD method [31]. Selection among MALDI-confirmed *Strep*. *uberis* strains was performed to enroll isolates from all selected herds. Thus, all isolates from herds with ≤9 strains that matched the inclusion criteria were selected for this study; whereas, for herds with ≥10 eligible strains, a random selection was performed using the RAND function of Excel software (2010; Microsoft Office Corporation, Redmond, WA, USA).

### RAPD typing

The genomic DNA was extracted from cultures without contamination using a commercial kit following the manufacturer’s recommendation (Illustra bacteria genomicPrep Mini Spin Kit, GE Healthcare, United Kingdom).

Bacterial DNA was amplified using primer OPE-04 (5’-GTGACATGCC-3’; Exxtend Solução em Oligos, Campinas, Brazil) as described by Wieliczko et al. [22] with some modifications: reactions contained 12.5 μL of Go Taq^®^ Green Master Mix 2x (Promega, Madison, WI, USA), 1.25 μL of primer OPE-04, 5 μL of genomic DNA (20 ng/μL), and sterile deionized water to a final volume of 25 μL. The PCRs were performed in a DNA Thermal Cycler (Eppendorf Mastercycler Gradient, Hamburg, Germany). Cycling included an initial denaturation step at 94°C for 2 min, followed by 39 cycles at 94°C for 30 s, 33°C for 30 s, and 72°C for 2 min. Ramp times were at 0.5°C/s. All isolates were analyzed in a single batch without repeating the assay.

All amplified products were electrophoresed at once in 2% agarose gel (96 wells) using TBE buffer (0.9 M Tris base, 0.09 M boric acid, 2.5 mM EDTA; pH 8.3) at 125 V for 55 min. The agarose gel was stained with *SYBR*^*™*^ *Safe DNA Gel Stain* (1:10,000; Thermo Scientific, Carlsbad, CA, USA). A photo-documentation system equipped with an ultraviolet light (Syngene, GeneGenius, Cambridge, United Kingdom) was used to capture gel images. A DNA ladder with 100-bp (Promega, Madison, WI, USA) was used for comparison with band sizes of *Strep*. *uberis* strains.

### Antimicrobial susceptibility testing

Antimicrobial susceptibility tests were performed using a commercial broth microdilution test specific for evaluation of mastitis pathogens (CMV1AMAF; Sensititre, TREK Diagnostics, Cleveland, OH, USA). The AMS testing was performed according to manufacturer’s instructions, which was detailed elsewhere [32]. Briefly, pure isolates were suspended in 0.9% saline solution to approximate the density of a 0.5 McFarland standard. Aliquots of 100 μL of bacteria were transferred to a tube containing 11 mL Mueller-Hinton broth (pH = 7.3 ± 1; BD, Sparks, MD, USA) supplemented with 5% of lysed horse blood. Sensititre panels were reconstituted with 50 μL of bacteria inoculum per wheel and incubated at 35°C for 20–24 h. After incubation, plates were read using the Sensititre Manual Viewer (TREK Diagnostic Systems, LLC, Cleveland, OH, USA) and the MIC was recorded as the lowest concentration of the antimicrobial that inhibited visible bacterial growth.

Table 2 presents the antimicrobials dilution ranges and interpretive criteria for susceptibility determination. Minimal concentrations that inhibited 50% (MIC_50_) and 90% (MIC_90_) of the isolates were recorded and their characterization as susceptible, intermediate or resistant was done according to guidelines of CLSI [33]. Strains with intermediate susceptibility to antimicrobials were classified as resistant. For ceftiofur, penicillin+novobiocin and pirlimycin, the susceptibility breakpoints were based on bovine mastitis data, according CLSI guidelines [33]. For the remaining antimicrobials (ampicillin, cephalothin, erythromycin, penicillin and tetracycline), the susceptibility categorization was based on human-derived breakpoints of viridans group streptococci or *Streptococcus* spp. when available (Table 2). Oxacillin is included in the commercial plate (CMV1AMAF; Sensititre) to test for methicillin-resistant *Staphylococcus aureus*, which was not of interest in the current study. Sulfadimethoxine dilutions also compose the plate, however, no breakpoints for this antimicrobial was found in the CLSI guidelines. Therefore, the results of *Strep*. *uberis* susceptibility to oxacillin and sulfadimethoxine were not reported in the present study.

**Table 2:** Dilution ranges and susceptibility breakpoints used in the antimicrobial susceptibility test of *Streptococcus uberis* isolated from CM.

Antimicrobial	MIC dilution range (μg/mL)	CLSI breakpoints (μg/mL)^1^
Ampicillin^2^	0.12–8.0	0.25
Ceftiofur^3^	0.5–4.0	2.0
Cephalothin^2^	2.0–16.0	8.0
Erythromycin^2^	0.25–4.0	0.25
Penicillin^2^	0.12–8.0	0.12
Penicillin+novobiocin^3^	1.0/2.0–8.0/16.0	1.0/2.0
Pirlimycin^3^	0.5–4.0	2.0
Tetracycline^2^	1.0–8.0	2.0

^1^Susceptibility breakpoints according to the guidelines of CLSI [33]. Isolates with minimum inhibitory concentrations less than or equal to the stated breakpoints were considered as susceptible to the antimicrobial.

^2^Human-derived susceptibility breakpoints. As no breakpoints were available for *Strep*. *uberis* recovered from bovine mastitis in the CLSI guidelines, the susceptibility to these antimicrobials was based on breakpoints of viridans group streptococci or *Streptococcus* spp.

^3^Susceptibility breakpoints based on bovine mastitis data.

### Data analysis

Descriptive data analysis, which included the severity score of CM [28] and distribution of RAPD-types within herds, was determined using the FREQ procedure of SAS 9.4 (SAS Inst. Inc., Cary, NC, USA).

**Fig 1:**
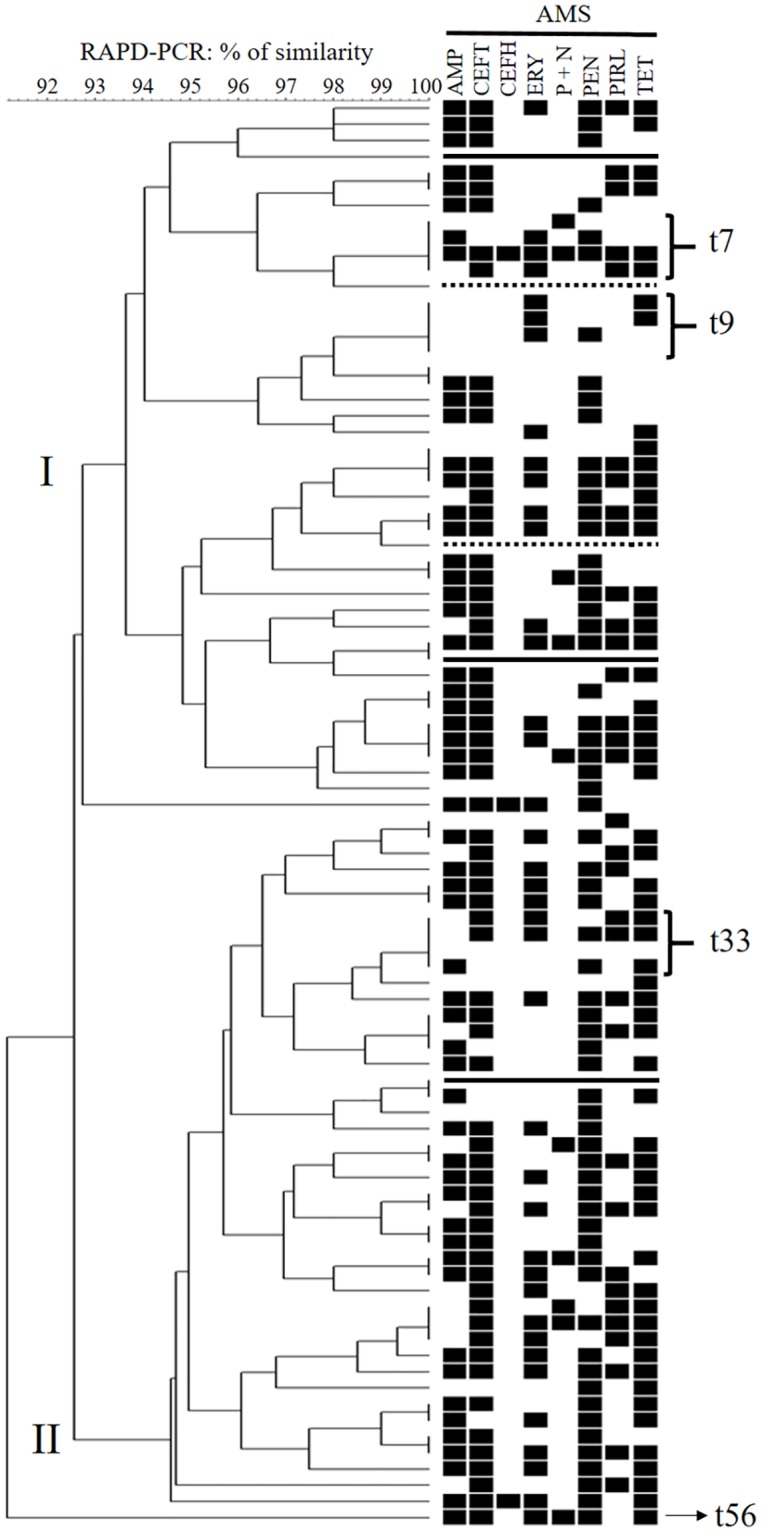
Dendrogram of RAPD profiles of *Strep*. *uberis* strains (n = 88) recovered from cows with clinical mastitis and results of susceptibility to 8 antimicrobials (n = 83). Three isolates were contaminated (solid line) and two had no growth during re-inoculation for AMS testing (dotted line). The interpretive criteria to categorize the RAPD-types as resistant (black boxes) or susceptible (without boxes) were based on CLSI guidelines [33].

The BioNumerics software v. 6.6 (Applied Maths, Sint-Martens-Ladem, Belgium) was used to construct a dendrogram based on the RAPD typing results. The resulting dendrogram was created by unweighted pair group method with arithmetic mean (UPGMA) using the default configuration with both position tolerance and optimization of 1%. Therefore, strains sharing the same number and the same sizes of PCR bands (i.e., 100% similarity) were considered genetically identical strains, while any relationship >90% and <100%, was defined as closely related, but not identical strains. RAPD-types were grouped into two clusters (I and II) based on the genetic similarity of isolates observed in the dendrogram (Fig 1).

The association between genotypic clusters and antimicrobial susceptibility (susceptible or resistant) was assessed in 8 separate mixed regression models (1 model for each antimicrobial tested) with binary distribution. To control for the potential effect of herd-level factors (e.g., drug use practices) on the AMS of *Strep*. *uberis* isolates, all models included the herd as random effect. Analyses were carried out using PROC GLIMMIX of SAS version 9.4 (SAS Inst. Inc., Cary, NC, USA). Statistical significance was assumed at *P* ≤ 0.05.

## Results

### Descriptive results and genotyping

All selected isolates were submitted for strain typing; however, one isolate (herd D) was not amplified during the RAPD-PCR and was excluded from the study. A high-level of polymorphism (56 RAPD-types) was observed among the remaining 88 fingerprinted isolates and all *Strep*. *uberis* isolates shared more than 90% of similarity based on the number and size of PCR bands (Fig 1). Only two cows had isolates recovered from repeated cases of CM, and the same RAPD-type was observed only for one of them, which may be due to the persistence of IMI.

Two clusters (I and II) were created according to the genetic similarity of RAPD-types (Fig 1). Cluster I was composed of 44 isolates and cluster II had 43 isolates. In addition, one isolate (named here as t56) had a lower level of similarity in comparison to other isolates and was not assigned into clusters. Cluster I had 28 RAPD-types, and the two most frequent types (t) were t7 and t9, with only four isolates each. Cluster II was composed of 27 RAPD-types, and the most frequent one was t33, with four isolates (Fig 1). Except for three herds (F, O and P), a unique cluster was predominant among isolates within herd (Table 3).

**Table 3:** Distribution of 88 *Streptococcus uberis* recovered from CM in dairy cows from 17 dairy herds according to RAPD-clusters, herd of origin, housing system, and severity score of clinical mastitis.

Variable	Categories	I		II		RAPD-type 56	
		n	%	n	%	n	%
Herd	A (n = 9)	9	100	-	-	-	-
	B (n = 2)	2	100	-	-	-	-
	D (n = 12)	12	100	-	-	-	-
	E (n = 15)	15	100	-	-	-	-
	F (n = 9)	1	11.1	8	88.9	-	-
	H (n = 5)	-	-	5	100	-	-
	I (n = 5)	-	-	4	80	1	20
	J (n = 2)	-	-	2	100	-	-
	K (n = 1)	-	-	1	100	-	-
	L (n = 16)	-	-	16	100	-	-
	M (n = 3)	3	100	-	-	-	-
	N (n = 1)	-	-	1	100	-	-
	O (n = 3)	1	33.3	2	66.7	-	-
	P (n = 2)	1	50	1	50	-	-
	Q (n = 1)	-	-	1	100	-	-
	R (n = 1)	-	-	1	100	-	-
	T (n = 1)	-	-	1	100	-	-
Housing^1^	Free stall (n = 26)	24	92.3	2	7.7	-	-
	CBPB (n = 11)	2	18.2	8	72.7	1	9.1
	Paddocks (n = 51)	18	35.3	33	64.7	-	-
Severity^2^	Mild (n = 50)	23	46.0	26	52.0	1	2.0
	Moderate (n = 34)	17	50.0	17	50.0	-	-
	Severe (n = 3)	3	100.0	-	-	-	-

^1^Housing system of herds from which the *Strep*. *uberis* isolates were selected. CBPB (Compost Bedded Pack Barn): housing system composed by a resting area bedded with organic materials, which must be mechanically stirred on a regular basis; Paddocks: housing system defined as an open area surrounded by fences or rails with or without pasture for grazing.

^2^CM severity was recorded as mild, moderate and severe according to Wenz et al. [28]. Of the selected isolates (n = 88), one did not have record of CM severity, which is not described in this variable

A total of 87 of 88 *Strep*. *uberis* isolates had record of CM severity: 50 (57.5%) were recovered from mild cases, 34 (39.1%) from moderate cases, and 3 (3.4%) from cases with severe signs (Table 3). For isolates recovered from mild cases, 23 (46.0%) were assigned to cluster I, 26 (52.0%) to cluster II, and one was the RAPD-type 56 (2.0%). For isolates recovered from moderate cases, 17 (50.0%) belonged to cluster I, and 17 (50.0%) belonged to cluster II. The three isolates identified from severe cases were assigned to cluster I.

### Overall antimicrobial susceptibility testing

Of 88 evaluated strains, three were excluded from AMS testing because of contamination, and two had no growth in the positive controls of the microdilution test. Therefore, 83 *Strep*. *uberis* strains had results of AMS. High rates of resistance were found to most antimicrobials, except for cephalothin and penicillin+novobiocin that showed higher susceptibility frequencies (≥88%; Fig 1; Table 4).

**Table 4:** Overall antimicrobial susceptibility of 83 strains of *Streptococcus uberis* isolated from clinical mastitis in 17 dairy herds from southeastern Brazil.

Antimicrobial	Frequency (%) of isolates at each indicated MIC (μg/mL)^1^												Res^2^	MIC_50_^3^	MIC_90_^4^
	0.12	0.25	0.5	1	2	4	8	16	32	64	128	256			
Ampicillin	18.1	15.7	51.8	3.6	0.0	1.2	1.2	-	-	-	-	-	8.4	0.5	4
Ceftiofur	-	-	8.5	4.8	9.6	41.0	-	-	-	-	-	-	36.1	4	>4
Cephalothin	-	-	-	-	88.0	6.0	2.4	2.4	-	-	-	-	1.2	2	4
Erythromycin	-	53.1	8.4	6.0	3.6	1.2	-	-	-	-	-	-	27.7	0.25	>4
Penic+Novob.	-	-	-	88.0	3.6	4.8	0.0	-	-	-	-	-	3.6	1	2
Penicillin	24.1	25.3	38.6	8.4	1.2	1.2	0.0	-	-	-	-	-	1.2	0.5	1
Pirlimycin	-	-	48.2	4.8	7.2	1.2	-	-	-	-	-	-	38.6	1	>4
Tetracycline	-	-	-	7.2	22.9	13.3	1.2	-	-	-	-	-	55.4	>8	>8

^1^The light gray shading represents the susceptible zone, and the darker gray shading represents the resistant zone. Results were interpreted according to CLSI [33]. Interpretive criteria were based on human data (ampicillin, cephalothin, erythromycin, penicillin and tetracycline), and bovine mastitis (ceftiofur, penicillin+novobiocin and pirlimycin). The resistant category included those isolates categorized as either intermediate or resistant.

^2^Res: proportion of isolates that were resistant at the highest antimicrobial concentration tested.

^3^MIC (μg/mL) that inhibited 50% (MIC_50_) of the isolates.

^4^MIC (μg/mL) that inhibited 90% (MIC_90_) of the isolates.

More than 50% of *Strep*. *uberis* were not inhibited at the highest concentration of tetracycline contained in the test. In relation to the MIC_90_ evaluation, in addition to tetracycline, more than 10% of *Strep*. *uberis* were not inhibited at the highest concentration of pirlimycin, ceftiofur, and erythromycin (Fig 1; Table 4).

### Antimicrobial susceptibility testing of genotypic clusters

The highest differences of AMS among clusters were found for tetracycline and penicillin. No other major differences in the proportions of resistant isolates were observed between clusters in the descriptive evaluation (Table 5). Furthermore, the logistic regression models showed no differences (*P >* 0.05) in AMS among clusters I and II.

**Table 5:** Antimicrobial susceptibility of 82 strains of *Streptococcus uberis* stratified according to their genetic similarity into RAPD-clusters I (n = 40) and II (n = 42). All isolates were recovered from cases of clinical mastitis in 17 dairy herds from southeastern Brazil.

Antimicrobial	Cluster	Frequency (%) of isolates at each indicated MIC (μg/mL)^1^												Res^2^	MIC_50_^3^	MIC_90_^4^
		0.12	0.25	0.5	1	2	4	8	16	32	64	128	256			
Ampicillin	I	22.5	7.5	47.5	7.5	0.0	0.0	2.5	-	-	-	-	-	12.5	0.5	>8
	II	14.3	23.8	54.8	0.0	0.0	2.4	0.0	-	-	-	-	-	4.7	0.5	0.5
Ceftiofur	I	-	-	12.5	10.0	2.5	42.5	-	-	-	-	-	-	32.5	4	>4
	II	-	-	4.8	0.0	16.7	38.1	-	-	-	-	-	-	40.4	4	>4
Cephalothin	I	-	-	-	-	90.0	5.0	0.0	2.5	-	-	-	-	2.5	2	2
	II	-	-	-	-	85.7	7.1	4.8	2.4	-	-	-	-	0.0	2	4
Erythromycin	I	-	57.5	12.5	5.0	5.0	0.0	-	-	-	-	-	-	20.0	0.25	>4
	II	-	50.0	4.8	7.1	2.4	2.4	-	-	-	-	-	-	33.3	0.25	>4
Penic+Novob.	I	-	-	-	87.5	5.0	5.0	0.0	-	-	-	-	-	2.5	1	2
	II	-	-	-	90.5	2.4	4.7	0.0	-	-	-	-	-	2.4	1	1
Penicillin	I	30.0	15.0	45.0	5.0	0.0	2.5	0.0	-	-	-	-	-	2.5	0.5	0.5
	II	19.1	35.7	33.3	9.5	2.4	0.0	0.0	-	-	-	-	-	0.0	0.25	1
Pirlimycin	I	-	-	45.0	5.0	10.0	2.5	-	-	-	-	-	-	37.5	1	>4
	II	-	-	50.0	4.8	4.8	0.0	-	-	-	-	-	-	40.4	0.5	>4
Tetracycline	I	-	-	-	7.5	30.0	12.5	0.0	-	-	-	-	-	50.0	4	>8
	II	-	-	-	7.1	16.7	14.3	2.4	-	-	-	-	-	59.5	>8	>8

^1^The light gray shading represents the susceptible zone, and the darker gray shading represents the resistant zone. Results were interpreted according to CLSI [33]. Interpretive criteria were based on human data (ampicillin, cephalothin, erythromycin, penicillin and tetracycline), and bovine mastitis (ceftiofur, penicillin+novobiocin and pirlimycin). The resistant category included those isolates categorized as either intermediate or resistant.

^2^Res: proportion of isolates that were resistant at the highest antimicrobial concentration tested.

^3^MIC (μg/mL) that inhibited 50% (MIC_50_) of the isolates.

^4^MIC (μg/mL) that inhibited 90% (MIC_90_) of the isolates.

## Discussion

Our study compared the AMS among RAPD clusters created according to the genetic similarity of *Strep*. *uberis* strains isolated from CM in 17 dairy herds of Southeastern Brazil. No differences in the resistance patterns were observed between clusters I and II for all antimicrobials evaluated. One potential cause for the lack of difference in the AMS among RAPD clusters may be the high frequency of genetically closely related strains according to the RAPD analysis. Based on the typing results, the number and sizes of PCR bands were >90% similar among the selected strains.

Despite the high level of genetic relationship between strains, 56 RAPD-types were identified among the 88 fingerprinted isolates. It is important to note that to be considered identical, strains had to present 100% similarity in the profile of PCR bands. The high polymorphism observed among strains in our study is in accordance with other reports [2, 4, 22], and is consistent with the hypothesis that the environment harbors a high diversity of *Strep*. *uberis* genotypes acting as the cause of IMI in dairy herds. After clustering, a higher predominance of closely related genotypes was observed within herds and housing system, especially in free stalls (24 of 26 isolates belonged to cluster I) and CBPB (8 of 11 isolates belonged to cluster II). Because of this high frequency of closely related strains within herds, it is reasonable to speculate that the CM caused by *Strep*. *uberis* in our study may originated from the exposure of the teat to a common environmental source, such as contaminated bedding material. However, this result must be interpreted with caution as most enrolled herds (i.e., 10 of 17) had ≤3 isolates submitted for genotyping. A higher frequency and distribution of isolates between herds could increase the polymorphism within herds.

Even with the low frequency of identical genotypes in our study, the hypothesis of potential transmission of *Strep*. *uberis* from cow-to-cow cannot be ruled out, as a high genetic similarity between isolates within herds was observed in our study. For example, of 15 *Strep*. *uberis* evaluated in herd E, four belonged to RAPD-type 7 and four to RAPD-type 9 (Table 3). Furthermore, the strains belonging to Cluster I were mainly found in herds A, B, D, E and M, while strains assigned to Cluster II were found mainly in other herds. Other studies evaluating *Strep*. *uberis* recovered from mastitis also reported closely related genotypes within and between herds, although different strain typing methods were used [3, 4, 34]. It is reasonable to suggest that herds with inadequate management practices for controlling contagious pathogens may facilitate the cow-to-cow transmission of certain *Strep*. *uberis* strains, even though the environment is the main reservoir of the pathogen [35].

RAPD was used in our study to characterize the genetic diversity of *Strep*. *uberis* isolates, although other methods (e.g., PFGE or MLST) were described as more discriminatory and easy-to-compare with results from other studies [17]. Using PFGE, other reports described higher level of polymorphism among *Strep*. *uberis* isolated from mastitis compared with our results [3, 4]. Therefore, one may speculate that the genetic similarity among genotypes could be lower if a more discriminatory strain typing method was used. For example, our results showed differences in the AMS among genotypes sharing the same RAPD-type (e.g., t7, t9, and t33; Fig 1), which suggests that these isolates may not be genetically identical as detected by the RAPD method. RAPD was chosen in our study because is a low-cost strain typing method, fast and easy to perform, and mainly because it presented sufficient discriminatory power for determination of *Strep*. *uberis* diversity in previous studies [2, 22]. However, we recognize that RAPD has poor reproducibility of fingerprints, and that minimal changes in the PCR conditions can lead to differences in the final results and comprise the ability to compare with other batches or other studies [36]. Therefore, all selected isolates were analyzed in a single batch to avoid potential variation that could happen if isolates were segregated in more than one batch of analysis. In addition, strains were considered identical only if they had 100% of genetic similarity.

Regardless of the genotypic profile, *Strep*. *uberis* strains were highly resistant to most antimicrobials evaluated in this study, including β-lactams. Over 50% of isolates were resistant to ampicillin, ceftiofur and penicillin. These results were not in accordance with studies evaluating AMS of *Strep*. *uberis* causing IMI in dairy cattle [35, 37–39]. For example, a high susceptibility of *Strep*. *uberis* was reported by Rossitto and coworkers [37], especially for β-lactams antimicrobials. However, comparisons between studies evaluating AMS may not be an easy task, especially due to differences among methods (disk diffusion or broth microdilution test), and because of the breakpoints used to categorize the pathogen as susceptible or resistant. In addition, there is limited data about the AMS of mastitis pathogens, especially for *Strep*. *uberis*.

The frequencies of AMS observed in the present study for cephalothin (97.2% *vs* 96.4% in our study), tetracycline (27.1% *vs* 30.1%), pirlimycin (60.9% *vs* 60.2%) and erythromycin (51.9% *vs* 53.1%) were similar to that reported by Rossitto and coworkers [37]. Moreover, MIC values found in our study were higher for all antimicrobials except for erythromycin and pirlimycin. Higher AMS to most evaluated antimicrobials were also reported in another study [40], excepting to penicillin and cephalothin, for which the susceptibilities were quite similar to ours. A noteworthy result in our study was the high resistance of *Strep*. *uberis* to penicillin, which is rarely reported in the literature [40, 41].

Differences in the susceptibility patterns among the published studies could be due to the different antimicrobial use in farms, regions or countries. A contemporary study characterizing the antimicrobials used for treatment of CM [8] in the same herds enrolled in this study, may suggest a potential relationship between the frequency of antimicrobial use and the high resistance of *Strep*. *uberis* isolates. For example, β-lactam antibiotics (e.g., penicillin and cephalosporins) and formulations containing tetracycline were compounds with high frequency of use in the aforementioned study, which also presented high proportion of resistance among *Strep*. *uberis* evaluated in the present study.

Identification of predominant strains causing infections such as mastitis in production animals, as well as periodic determination of antimicrobial susceptibility profile, can provide the opportunity to better understand the epidemiology and determine the resistance profile of pathogenic bacteria in a given geographical area. However, a recognized limitation about the evaluation of AMS of mastitis-causing pathogens is the lack of bovine-specific interpretive criteria to categorize isolates as resistant or susceptible. Most cut-off values used for evaluation of pathogens causing bovine mastitis are still based on other animal species or human interpretive criteria, except for pirlimycin, penicillin+novobiocin, and ceftiofur [33]. Under these circumstances, the clinical outcome after bovine mastitis treatment cannot be predicted with accuracy using the current breakpoints. Microdilution test for evaluation of MIC can be more accurate for resistance profile monitoring than simply classify bacteria as resistant or susceptible based on nonspecific breakpoints. The evaluation of MIC results from different geographical areas could enable further studies to establish a system to interpret the AMS of mastitis pathogens in dairy cattle using specific breakpoints.

Even with the high polymorphism observed among *Strep*. *uberis* isolated from CM in our study, the RAPD results allowed us to create two clusters based on the genetic similarity between strains, although no significant differences in the susceptibility to any antimicrobials tested were found between clusters. These results may be associated with the high overall AMR observed among the *Strep*. *uberis* selected in our study, which could be a consequence of antimicrobial overuse for treatment of CM in Brazilian dairy herds [8]. Further studies should be conducted to determine the potential association between antimicrobial use and the resistance of *Strep*. *uberis* causing CM in dairy herds of Brazil.

## Conclusion

Molecular analysis using RAPD showed high polymorphism among *Strep*. *uberis* isolated from CM, whereas a higher frequency of closed-related strains was observed within herds after clustering. Regardless of strain typing results, *Strep*. *uberis* were categorized as resistant to most antimicrobials, except to cephalothin and penicillin+novobiocin. Although some differences in the proportions of MIC_50_ and MIC_90_ were observed among genotypic clusters, mixed regression models showed no differences between them for all antimicrobials evaluated. Since RAPD has reproducibility drawback and is not recognized as a highly discriminatory method, our results may not be comparable to other molecular studies evaluating *Strep*. *uberis* causing bovine IMI.
